# Preparation of long afterglow luminescent road marking coatings and its applicability simulation

**DOI:** 10.1371/journal.pone.0330387

**Published:** 2025-09-15

**Authors:** Guojun Li, Ning Li, Yan Zhang, Jie Han, Tengfei Yao, Xiaowei Feng

**Affiliations:** 1 Gansu Province Traffic Investment Management Co., Ltd., Lanzhou, China; 2 Gansu Industry Technology Center of Transportation Construction Materials Research and Application, Lanzhou Jiaotong University, Lanzhou, Gansu, China; 3 Gansu-highway Traffic Construction Group Co., Ltd, Lanzhou, Gansu, China; 4 Gansu Provincial Transportation Research Institute Group Co., Ltd, Lanzhou, Gansu, China; Monash University, AUSTRALIA

## Abstract

Traffic markings have inadequate nighttime visibility for safe driving. To address this and reduce nighttime accidents, we researched and developed long afterglow luminescent coatings, including performance and simulation studies. By varying the dosage of luminescent powder, glass powder, and titanium dioxide, nine groups of coatings were fabricated via blending. Their luminescence and chromaticity properties were investigated. Using CW-VIKOR comprehensive evaluation, the optimal formulation was selected. This optimal coating was further analyzed using Twinmotion and DIALux simulations, assessing marking visibility and tunnel illumination effects with the coatings.Results revealed that increased luminescent powder dosage enhanced luminescence performance. The optimal formulation (30% luminescent powder, 8% glass powder, 4% titanium dioxide) achieved the best luminescence performance and colorimetric characteristics, sustaining light emission for 9 hours with good road performance. Twinmotion simulations demonstrated better nighttime visibility at different brightness levels. DIALux simulations showed a 22.3% increase in average tunnel brightness after coating deployment, significantly improving lighting effects.

## 1 Introduction

In recent years long afterglow luminescent materials are gradually used in road construction. The materials absorb natural light or lighting sources and slowly release the stored energy in the form of light after stopping the irradiation of the light source, realizing non-electrical luminescence [1–3]. Luminescent coatings made from luminescent materials are widely used in road signs, warnings, tourist areas, and inside tunnels [4,5]. They facilitates drivers to recognize road conditions under extremely adverse conditions such as rain, fog, and darkness, reduces the incidence of traffic accidents, and ensures driving safety.

Currently, there are strontium aluminate luminous powder in traffic marking applications. Jason Nance et al. [6,7] investigated the role of rare-earth doped strontium aluminate instead of glass beads, which still emits light stably and acts as an inducer and a warning when it is not exposed to incident light. Gutierrez et al. [8] demonstrated the feasibility of strontium aluminate luminescent powder in traffic marking, which can be a good solution to the problem of difficult recognition of traffic markings at night and under no illumination conditions. Cao et al. [9] prepared high luminescence long afterglow materials by the high-temperature solid phase reaction method. When the pigment-to-binder ratio is 0.50 and the long afterglow material accounts for 40%, the luminescent coating can continue to emit light for more than 7 hours in a dark environment. Bi et al. [10] prepared waterborne acrylic luminescent road marking paint with self-made modified SrAl_2_O_4_: Eu^2+^, Dy^3+^ as luminescent materials. The paint not only has good luminescent properties but also has good adhesion. There are more studies on the luminescent properties of luminescent materials, but fewer research results are on the ground. Therefore, this study uses analog simulation to verify the applicability of luminous markers.

With the rapid development of digital technology at home and abroad in recent years, virtual technology has gradually been applied to various industries. For billboards or road light-emitting markers that emit light on the outside of the building, etc., the light-emitting parameters can be adjusted for lighting design using material settings on the 3D model [11]. Although different parameters can be adjusted, they can only be used for qualitative analysis. Liang [12] carried out performance tests of reflective storage materials. It was found that with reflective storage materials in tunnels, the diffuse reflectance can be as high as about 80%, significantly improving the roadway brightness. Multifunctional light-storing luminescent coatings are used for tunnel auxiliary lighting with a luminous duration of 8 hours. Using these coatings improves the brightness and uniformity of the tunnel road surface [13]. DIALux is used to construct a three-dimensional model for tunnel lighting analysis, which can be used to calculate the average brightness, uniformity, and other indicators. To improve the quantitative analysis level of lighting design and reduce the operation’s energy consumption [14]. The average illumination of the tunnel side wall with luminescent coating was 12.8% higher than that of the fire retardant coating. It can improve the traveling environment in tunnels and reduce accidents [15].

Therefore, in this paper, a long afterglow luminescent coating was prepared by the blending method. The dosage of luminescent materials, glass powder, and titanium dioxide were considered to study their effects on the luminescence and chromaticity properties of the long afterglow luminescent coating. The optimal formulation of the luminescent coating was determined by combining the CW-VIKOR comprehensive evaluation. The luminescent coatings are also simulated and analyzed by Twinmotion and DIALux. Explore the visibility of luminescent marking and the illumination effect when the luminescent coating is deployed in tunnels.

## 2 Materials and methods

### 2.1 Materials

#### (1) Luminescent material.

The luminescent material is the key component of the long afterglow luminescent coating, which gives the luminescent properties to the long afterglow luminescent coating. In this paper, strontium aluminate was selected as the luminescent material, and the composition and characteristics of the luminescent material are shown in Table 1.

**Table 1:** Composition and characteristics of luminescent materials.

Luminescent material	Essential component	Afterglow color	Afterglow time/h	Characteristic
aluminate	SrAl_2_O_4_:Eu^2 + ^, Dy^3+^	yellowish green	10	high luminescent brightness, not easy to hydrolyze

#### (2) Film-forming substance.

Film-forming substances can adhere to other substances and form a thin film, one of the basic components of long afterglow luminescent coatings. According to the luminescence characteristics and application scenarios of long afterglow coatings, silicone-acrylic emulsion film-forming material was selected as long afterglow coatings’ main film-forming material. The physical properties of the film-forming substances are shown in Table 2.

**Table 2:** Physical properties of film-forming substances.

Film-forming substances	Appearance	pH value	Viscosity/mPa·s	Solid content/%
silicone-acrylic emulsion	bluish white emulsion	8	600	47 ± 1

#### (3) Pigments and fillers.

Pigments can improve the brightness of luminescent coatings. Rutile titanium dioxide is a highly reflective pigment, so rutile titanium dioxide was chosen as the pigment. Fillers improve the dispersion of coatings. Glass powder can not only enhance the dispersion of the coating, but at the same time, glass powder has better light transmission and reflective properties. Therefore, glass powder was chosen as the filler. The physical properties of the pigments and fillers are shown in Table 3.

**Table 3:** Physical properties of pigments and fillers.

Materials	Essential component	Appearance and color	Density/g ∙ cm^-3^	Mohs hardness
rutile titanium dioxide	TiO_2_	White powder	4.2	6.5
glass powder	SiO_2_	White powder	2.61	7.8

#### (4) Additive.

The addition of additives can improve the properties of coatings such as storage stability, workability, and leveling. There are more types of additives and different additives can improve different properties. In this paper, film-forming additives, anti-settling agents, leveling agents, and defoamers were selected to improve the performance of coatings. The film-forming additive component is alcohol ester twelve. The leveling agent adopts a water-based leveling agent. The defoaming agent is a silicone defoaming agent. Anti-settling agent using fumed silica. The physical properties of each additive are shown in Table 4.

**Table 4:** Physical properties of the additives.

Materials	Appearance and color	pH value	Viscosity/mPa·s	Solid content/%
film-forming additive	colorless transparent liquid	–	–	≥99.5
leveling agent	colorless to light yellow liquid	6	800	≥99.8
defoaming agent	milky white liquid	6.5	500	≥99
anti-settling agent	white powder	7	–	≥98

### 2.2 Preparation of luminescent coatings

The preparation of long afterglow luminescent coatings was carried out by blending method. The preparation process is as follows:

(1) Luminescent powder, pigmented filler, and fumed silica were weighed according to the proportion. At the same time, the silicone-acrylic emulsion was weighed and added to the beaker for stirring. Add half of the mass of fumed silica to the silicone-acrylic emulsion and stir for 10–15 min.
(2) The remaining half of the fumed silica was uniformly mixed with the luminescent material, pigments, and fillers. Add slowly and evenly, stirring for 25–30 minutes to form a homogeneous mixture.
(3) Add an appropriate amount of defoaming agent, leveling agent, and film-forming additives to the mixture, and continue to stir for 5–10 min. Finally, a light white coating is obtained, which was a long afterglow luminescent coating.

The specimens were prepared according to the orthogonal test design table in Table 5. Among them, are the dosage of strontium aluminate luminescent powder (factor A), the dosage of glass powder (factor B), and the dosage of titanium dioxide (factor C). The silicone-acrylic emulsion is used as a film-forming substance and is not used as a variable factor. A large variety of additives is also not a variable factor. Recommended dosage: anti-settling agent 0.6%, film-forming additive 2.4%, defoaming agent 0.8%, leveling agent 0.8%. The paint was applied to the cemented asbestos with a thickness of 600 μm using a paint applicator.

**Table 5:** Orthogonal test design table.

Test number	A/%	Vacant column	B/%	C/%
No.1	20	1	4	2
No.2	20	2	6	4
No.3	20	3	8	6
No.4	25	1	6	6
No.5	25	2	8	2
No.6	25	3	4	4
No.7	30	1	8	4
No.8	30	2	4	6
No.9	30	3	6	2

### 2.3 Test methods

#### (1) Brightness.

The brightness was tested by a CS-200 color luminance meter. The excitation light source is a D65 standard light source with an illuminance of 1000 lx. The excitation light source was directly irradiated on the surface of the luminescent material or luminescent coating for 20 min. Test the initial brightness as well as the 10 minute brightness. Before the test measurements, the prepared test plates need to be stored in a dark room, protected from light for 24 h.

#### (2) Afterglow time.

The afterglow time is the time from when the excitation source is turned off to when the luminous brightness drops to 0.32 mcd/m^2^ [16]. In this paper, from the light source, the human eye can not recognize the duration of the time that is identified as afterglow time, afterglow time recorded in hours.

#### (3) Brightness factor.

The brightness factor of coatings was tested with SP62 spectrophotometer. Place the sample under test on a flat table. The target window of the instrument is then placed on the sample and the instrument head is lowered for measurement. Three measurements were taken and averaged.

### 2.4 Simulation

DIALux has major advantages in terms of self-modeling, compatibility, illuminance calculation, and natural light analysis. DIALux can simulate the influence of sunshine and other conditions. It is a comprehensive and powerful light environment simulation software [17,18]. DIALux calculates indoor lighting, outdoor daylighting, and street lighting. The results are presented in a variety of ways including tables, pseudo-color plots, and iso-illumination plots. In addition, it is possible to use DIALux to set material parameters for imported or self-built models to change the lighting effect. DIALux has demonstrated excellent reliability and accuracy in tunnel lighting. The simulated tunnel lighting effect almost mirrors the actual lighting effect. The relative error between measured and simulated illuminance values is controlled within 10%, providing support for tunnel lighting design [19].

To compare the enhancement of pavement brightness after luminous paint deployment, three types of sidewall interior materials were considered: ordinary fireproof paint, reflective tiles, and luminous paint. Ordinary fireproof coating is consistent with the roof material, the reflection ratio is 0.10. Reflective tiles are laid for 3 m, the reflection ratio is 0.58. Luminous coatings are mainly reflected in the software with high reflection ratio, and the minimum reflection ratio of luminous coatings under normal conditions is 0.85, which is taken as 0.85 in this paper.

## 3 Results and discussion

### 3.1 Performance study of long afterglow luminescent coatings

**Fig 1:**
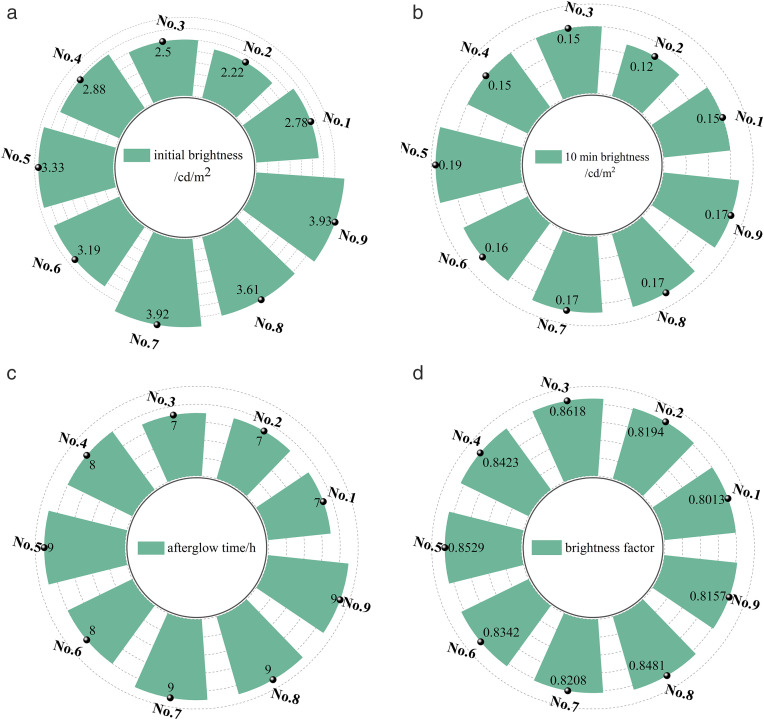
Luminescence and colorimetric characteristics of luminescent coatings. (a) Initial brightness (b) 10 min brightness (c) Afterglow time (d) Brightness factor.

Currently, there are many indexes for evaluating the luminescent performance of luminescent coatings, including brightness, colorimetric, luminous flux, afterglow time, excitation spectrum, and emission spectrum [20]. In this paper, the initial brightness, 10 min brightness and afterglow time are used as evaluation indexes in combination with the actual situation. The colorimetric characteristics of the coating should also be considered, taking the brightness factor as the evaluation index [21]. The test results of the long afterglow luminescent coatings are shown in Table 6 and Fig 1.

**Table 6:** Test results of long afterglow luminescent coatings.

Test number	Initial brightness(cd/m^2^)	10 min brightness(cd/m^2^)	Afterglow time(h)	Brightness factor
No.1	2.78	0.15	7	0.8013
No.2	2.22	0.12	7	0.8194
No.3	2.5	0.15	7	0.8618
No.4	2.88	0.15	8	0.8423
No.5	3.33	0.19	9	0.8529
No.6	3.19	0.16	8	0.8342
No.7	3.92	0.17	9	0.8208
No.8	3.61	0.17	9	0.8481
No.9	3.93	0.17	9	0.8157

Brightness is regarded as a characteristic of color, which is used to describe the subjective bright feeling perceived by the human eye. The unit is cd/m^2^ or nits. But not every product is a good product if it is brighter, and the criteria for judging it are closely related to human vision. If the monitor screen is too bright, it is easy to produce visual fatigue and make the viewer feel uncomfortable. This same pattern is followed when using illuminated markers. Excessive brightness will cause people’s visual fatigue. The brightness is low, which cannot guide the night vision well, and it is difficult to ensure the safety of night travel. Only the appropriate brightness can better guide the line of sight. According to the CIE, there are three types of vision in the human eye: bright vision, mesopic vision and dark vision. Bright vision is usually specified above 5 cd/m^2^ and is dependent on cone cell action. Dark vision is below 10^-3^ cd/m^2^, and it is the optic rod cells that play a role. The brightness of mesopic vision is between bright vision and dark vision, and the brightness range is 10^-3^ ~ 5 cd/m^2^, which is coordinated by cone cells and rod cells [22]. Relevant research shows that when the human eye is in the middle of vision, the visual effect is the best. In life, lighting for mesopic vision is more common, such as roadway lighting, tunnel lighting, airport lighting, emergency lighting, etc [23,24]. The brightness of the road luminous markings prepared in this paper is below 5 cd/m^2^, which is in the brightness range of mesopic vision.

From Fig 1(a), it can be seen that the initial brightness of the luminescent coating shows an increasing trend with the increase of luminescent powder dosage. Among them, the maximum brightness is No.9, and the brightness is 3.93 cd/m^2^. As can be seen from Fig 1(b), the 10 min brightness change is small. Meanwhile, the 10 min luminance was all greater than the normative value of 0.05 cd/m^2^. From Fig 1(c), it can be seen that the afterglow time also shows an increasing trend with the increase of luminescent powder dosage. The luminous brightness and afterglow time increase with the increase of luminescent powder dosage. This is because luminescent powder is the main substance that makes luminescent paints glow. With the increase of the amount of luminescent powder, the luminescent materials per unit area will increase. Luminescent materials clustered together increase the number of luminescent centers, further increasing luminous brightness and afterglow time. As can be seen in Fig 1(d), the luminance factor of all groups of coatings is greater than 0.8. The brightness factor is mainly affected by the dosage of titanium dioxide, and it can be seen that the brightness factor increases with the increase of titanium dioxide dosage [25]. The magnitude of the luminance factor is determined by the surface properties and reflectivity of the material itself. Among them, titanium dioxide belongs to high reflectivity pigments, and high reflectivity can increase its brightness factor.

### 3.2 Performance evaluation of coatings based on CW-VIKOR

#### 3.2.1 Determination of indicator weights.

In this study, the combined weights method of CRITIC and analytic hierarchy process were used to determine the index weight. It not only considers the subjective experience of experts but also considers the objective authenticity of the data so that the obtained index weight is more scientific and accurate.

(1) CRITIC determines objective weights

Diakoulaki et al. proposed the CRITIC method in 1995, which is an objective weighting method. The core of the methodology is to synthesize the degree of comparison of the evaluation indicators and the conflicting nature of the indicators in order to make a comprehensive assessment. Specifically, it determines the weights by calculating the standard deviation and correlation coefficient of the evaluation indicators. Both variability and correlation among indicators are taken into account [26]. The specific calculations are as follows:

1) Normalized processing

$$x_{ij}=\frac{b_{ij}}{\sqrt{\sum_{i=1}^{n}b_{ij}^{2}}}$$ (1)

Where $x_{ij}$ is the assessed value of the jth evaluation indicator of the standardized ith evaluation object, and $b_{ij}$ is the raw data of the jth evaluation indicator of the ith evaluation object. The standardized treatment results are shown in Table 7

**Table 7:** Standardized treatment.

Test number	Initial brightness(cd/m^2^)	10 min brightness(cd/m^2^)	Afterglow time(h)	Brightness factor
No.1	0.289348584	0.312567839	0.286012246	0.320588039
No.2	0.231062538	0.250054271	0.286012246	0.327829576
No.3	0.260205561	0.312567839	0.286012246	0.344793176
No.4	0.299756806	0.312567839	0.326871139	0.336991521
No.5	0.346593807	0.395919263	0.367730031	0.341232421
No.6	0.332022296	0.333405695	0.326871139	0.333750833
No.7	0.40800232	0.354243551	0.367730031	0.328389695
No.8	0.37573683	0.354243551	0.367730031	0.339312013
No.9	0.409043142	0.354243551	0.367730031	0.326349262

2) Variability of indices

$$\sigma_{j}=\sqrt{\frac{\sum {\mathrm{\backslash nolimits}}_{i=1}^{n}{\left(x_{ij}-{\bar{x}}_{j}\right)}^{2}}{n-1}}$$ (2)

$${\bar{x}}_{j}=\frac{1}{n}\sum {\mathrm{\backslash nolimits}}_{i=1}^{n}x_{ij}$$ (3)

where ${\bar{x}}_{j}$ is the average value of the indicators in the n evaluation objects and $\sigma_{j}$ is the standard deviation of the jth index.

3) Conflict of index indicators

$$R_{j}=\sum_{i=1}^{p}\left(1-r_{ij}\right)$$ (4)

The form of correlation coefficients was used to show the conflict of indicators. Where *r*_*ij*_ represents the correlation coefficient between evaluation indicators *i* and *j*.

4) Information content

$$C_{j}=\sigma_{j}\sum_{i=1}^{p}\left(1-r_{ij}\right)$$ (5)

Where $C_{j}$ is the amount of information contained in the jth indicator.

5) Objective weights

$${\hat{w}}_{j}=\frac{C_{j}}{\sum {\mathrm{\backslash nolimits}}_{j=1}^{p}C_{j}}$$ (6)

where ${\hat{w}}_{j}$ is the objective weight of the jth indicator. The objective weight calculation results of this study are presented in Table 8.

**Table 8:** Results of objective weight calculation.

Item	Variability of indices	Conflict of indicators	Information content	Weight/%
Initial brightness(cd/m^2^)	0.06314	1.38281	0.08731	44.914
10 min brightness(cd/m^2^)	0.04095	1.07932	0.04419	22.734
Afterglow time(h)	0.03792	1.10168	0.04177	21.487
Brightness factor	0.00798	2.64816	0.02112	10.865

(2) Determining subjective weight by analytic hierarchy process

The analytic hierarchy process (AHP) is a decision analysis method that combines qualitative and quantitative methods to solve complex multi-objective problems. Construct a hierarchical relationship diagram based on the correlation between decision objectives, decision factors and decision options. Make a subjective judgment of the relative importance between two indicators based on certain objective realities and construct a judgment matrix. Through the consistency test of the judgment matrix and the relevant numerical operations, the weights of each indicator can be obtained [27]. The steps to determine the subjective weight are as follows:

1) Establish a hierarchical structure model

**Fig 2:**
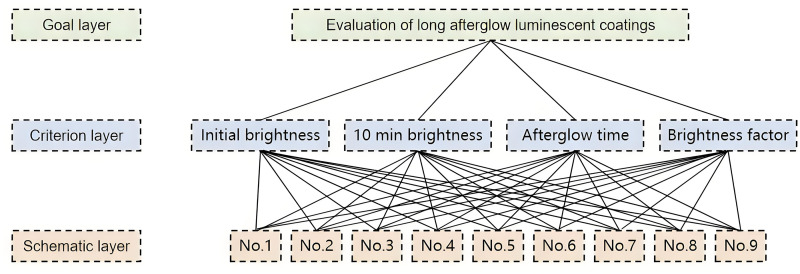
Hierarchical relationship diagram.

By constructing evaluation indicators to quantitatively evaluate the nine groups of coatings, the constructed hierarchical relationship diagram is shown in Fig 2.

2) Experts compare the indicators two by two and construct a judgment matrix *R*=(*A*_*ij*_)_*mxn*_

The elements have different strengths for each indicator at each coefficient level, and the strengths of the elements and the impacts of each sub-goal on the higher-level factors are also different. For the categorization of indices, the principle is to reflect the importance of the factors associated with that level by constructing an evaluation matrix. Quantification was performed using the 1–9 scale method and the quantified values are shown in Table 9.

**Table 9:** Scale quantization value description.

Relative importance	Definition
1	Compared with the two factors, factor i and j are equally important
3	Compared with the two factors, factor i and j are slightly important
5	Compared with the two factors, factor i and j are more important
7	Compared with the two factors, factor i and j are far more important
9	Compared with the two factors, factors i and j are extremely important
2, 4, 6, 8	an intermediate value of two adjacent judgments
1/2, 1/3, ..., 1/9	the importance of j to i is the reciprocal of the importance of i to j

3) Determining the eigenvectors of the judgment matrix R based on the square root method

Compute the nth root of the product of the elements of each row of the judgment matrix R. Equation (7) is given below:


$$M_{i}=n\sqrt{\prod {\mathrm{\backslash nolimits}}_{j=1}^{n}a_{ij}}$$ (7)

Normalizing $M_{i}$, Equation (8) is as follows:


$$W_{i}=\frac{M_{i}}{\sum {\mathrm{\backslash nolimits}}_{i=1}^{n}M_{i}}$$ (8)

Calculate the maximal characteristic root of the judgment matrix, Equation (9) as follows:


$$\lambda =\sum_{i=1}^{n}\frac{{\left(A\bar{\omega }\right)}_{i}}{n{\bar{\omega }}_{i}}$$ (9)

4) Consistency test on the judgment matrix R

If the consistency index *CR* < 0.1, R is considered to have good consistency, otherwise it needs to be corrected.


$$CI=\frac{\lambda -n}{n-1}$$ (10)

$$CR=\frac{CI}{RI}$$ (11)

*CI* is a measure of the judgment matrix deviation from the consistency index, when the CI is larger, the judgment matrix consistency is worse. On the contrary, when *CI* is 0, the judgment matrix is completely consistent. *CR* is the consistency ratio, which is calculated by *CI* and *RI*. Where *RI* is the average random consistency index, the value of *RI* can refer to Table 10. When *CR* < 0.1, the consistency of the judgment matrix can be considered acceptable. The results of subject weighting and results of the consistency test in this study are shown in Table 11 and 12, respectively.

**Table 10:** Average random consistency index.

n	1	2	3	4	5	6
*RI*	0	0	0.528	0.882	1.108	1.247
n	7	8	9	10	11	12
*RI*	1.339	1.404	1.451	1.486	1.514	1.536

**Table 11:** Results of subjective weighting.

Item	Eigenvector	Weight/%	Maximal characteristic root	CI value
Initial brightness(cd/m^2^)	0.98094	24.039	4.008	0.003
10 min brightness(cd/m^2^)	0.78012	19.118		
Afterglow time(h)	1.35540	33.216		
Brightness factor	0.96411	23.627		

**Table 12:** Results of the consistency test.

Maximal characteristic root	*CI* value	*RI* value	*CR* value	Consistency test results
12.05	0.003	0.882	0.003	pass

(3) Multiplication normalization method to determine the combined weight

The weights obtained from CRITIC method and AHP were combined using multiplicative normalization [28]. Equation (12) was used to perform the calculation to obtain the combined weights, and the combined weight values are shown in Table 13.

**Table 13:** Combined weight values.

Item	objective weights/%	subjective weights/%	combined weights/%
Initial brightness(cd/m^2^)	44.914	24.039	43.453
10 min brightness(cd/m^2^)	22.734	19.118	17.491
Afterglow time(h)	21.487	33.216	28.724
Brightness factor	10.865	23.627	10.331


$$\omega_{j}=\frac{{\bar{\omega }}_{j}{\hat{\omega }}_{j}}{\sum_{j=1}^{m}{\bar{\omega }}_{j}{\hat{\omega }}_{j}}$$ (12)

#### 3.2.2 VIKOR comprehensive evaluation.

The VIKOR method is an eclectic multi-criteria decision-making method proposed by Opricovic and Tzeng et al [29,30]. The basic idea of the VIKOR method is to determine positive and negative ideal solutions based on the optimized compromise solution method. The optimal program is then selected based on the proximity of the assessed value of each evaluation program to the ideal program. The specific process is as follows:

1) Dimensionless processing

$$f_{ij}=\frac{x_{ij}}{\sqrt{\sum_{i=1}^{n}x_{ij}^{2}}}$$ (13)

2) Determining the positive ideal solution and negative ideal solution

$$f_{j}^{*}=\left[\left(\underset{i}{\max}f_{ij}|j\in X\right),\left(\underset{i}{\min}f_{ij}|j\in Y\right)\right]$$ (14)

$$f_{j}^{-}=\left[\left(\underset{i}{\min}f_{ij}|j\in X\right),\left(\underset{i}{\max}f_{ij}|j\in Y\right)\right]$$ (15)

3) Calculate the group utility value and individual regret value of the evaluation object of VIKOR.

$$S_{i}=\sum_{j=1}^{m}\left(w_{j}\frac{f_{j}^{*}-f_{ij}}{f_{j}^{*}-f_{j}^{-}}\right)$$ (16)

$$R_{i}=\underset{j}{\max}\left(w_{j}\frac{f_{j}^{*}-f_{ij}}{f_{j}^{*}-f_{j}^{-}}\right)$$ (17)

4) Calculate the benefit ratio of each evaluation object.

$$Q_{i}=v\left(\frac{S_{i}-S_{i\min}}{S_{i\max}-S_{i\min}}\right)+\left(1-v\right)\left(\frac{R_{i}-R_{i\min}}{R_{i\max}-R_{i\min}}\right)$$ (18)

Whereν is the decision mechanism coefficient. ν >0.5 indicates that decisions are based primarily on maximizing group benefits. ν <0.5, indicating that the decision is based primarily on a small number of opposing views. ν =0.5, indicating that both group benefits and individual regrets are taken into account, andν =0.5 is chosen in this paper.

The positive ideal solution and negative ideal solution are given in Table 14, and the calculation results are given in Table 15. The calculation of the profit ratio combines the group utility value and the individual regret value. Through the decision mechanism coefficient, the VIKOR method can maximize the group utility and minimize individual regret. Smaller values of the benefit ratio indicate better results. It can be seen from Table 15 that the minimum benefit ratio of No.7 is 0, indicating that the brightness and colorimetric characteristics of No.7 are good. The maximum benefit ratio of No.2 is 1. The results were ranked based on the benefit ratio, with the rankings being No. 7, No. 9, No. 8, No. 5, No. 6, No. 4, No. 1, No. 3, and No. 2. Among them, No.7 is ranked first, which indicates that No.7 has better performance. Group No.7 coating has high brightness, long afterglow time, and good visibility. That is, when the dosage of luminescent powder is 30%, the dosage of glass powder is 8%, and the dosage of titanium dioxide is 4%, the luminescent coating has the best luminescence performance and colorimetric characteristics. For the best performing No.7 a comprehensive performance test was conducted and the results are shown in Table 16.

**Table 14:** Positive ideal solution and negative ideal solution.

Item	Positive ideal solution	Negative ideal solution
Initial brightness(cd/m^2^)	0.409043142	0.231062538
10 min brightness(cd/m^2^)	0.395919263	0.250054271
Afterglow time(h)	0.367730031	0.286012246
Brightness factor	0.344793176	0.320588039

**Table 15:** Calculation results and comprehensive ranking.

Test number	Group utility value	Individual regret value	Benefit ratio	Ranking
No.1	0.782734191	0.292227936	0.694739518	7
No.2	0.969091503	0.434530236	1	9
No.3	0.75057241	0.363379086	0.773340577	8
No.4	0.543688356	0.266816811	0.518697166	6
No.5	0.16766485	0.152466749	0.139756633	4
No.6	0.453758011	0.188042324	0.35752868	5
No.7	0.122530507	0.070013721	0	1
No.8	0.154686102	0.0813156	0.034494455	3
No.9	0.128698418	0.078722745	0.015588916	2

**Table 16:** Comprehensive performance test results.

Test items	Specified requirement	Test result
Condition in container	Easy to stir without caking	Easy to stir without caking
Coating Appearance	Leveling	Leveling
Water resistance	Immersion for 24 h without abnormalities	No abnormality
Alkali resistance	Immersion for 24 h without abnormalities	No abnormality
Freeze-thaw stability	No stratification phenomenon	No stratification phenomenon
Non-stick tire drying time	≤15 min	11 min
Slip resistance	≥45 BPN	74 BPN
Abrasion resistance	<60 mg	8.2 mg
Adhesion	≥1.5 MPa	2.53 MPa

It can be seen from Table 16 that the long afterglow luminescent marking coating prepared in the laboratory has excellent performance. The stability of the coating is good, the non-stick tire drying time is short, can meet the actual application requirements. At the same time, water resistance, alkali resistance, slip resistance, abrasion resistance, and adhesion are in line with and higher than the specification requirements. It shows that the luminescent coating has good durability.

### 3.3 Twinmotion visual simulation

**Fig 3:**
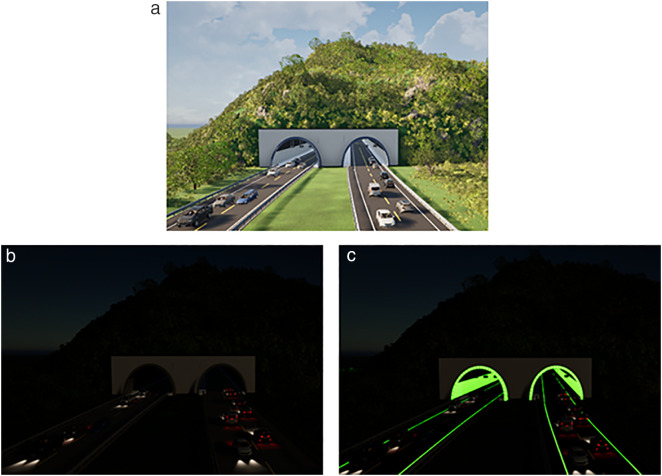
Tunnel model under different lighting conditions. (a) Daytime tunnel model (b) Tunnel model without lighting facilities at night (c) Tunnel model with luminous coating applied to unlit sections at night.

The brightness of the prepared luminescent coating belongs to the brightness range of mesopic vision, that is, the luminescent coating can be used for road lighting, tunnel lighting, airport lighting, and emergency lighting. To better present the visualization effect of the coating, the luminescent coating is applied to the road markings in the tunnel and the side wall of the tunnel. Due to the best luminescence and colorimetric characteristics of No. 7, the tunnel model was simulated with No. 7 group coating data. Twinmotion was used for simulation. The simulation conditions were: sunny day, wind speed of grade 1, wind direction of 10°, and vegetation growth of 0.5. Ambient light 1.0, contrast 50%, saturation 50%, the remaining environmental conditions are the default conditions. The tunnel models in different states are shown in Fig 3.

Fig 3 illustrates the tunnel model at different states. Fig 3(a) shows the tunnel model for daytime hours, with a two-bore unidirectional traffic setup, vegetation as well as vehicles, at 8:00 am. With the ordinary markings, the entrance section is displayed, and the specific environment in the tunnel is not described. Fig 3(b) shows the scene at the tunnel entrance at night when there is no lighting or when the lighting fails, and the sidewalls are covered with ordinary fireproof coating. At the entrance of the tunnel, only the car lights can be seen, and it is impossible to see whether the inside of the tunnel is a straight line or a curved line. It affects the human eye’s recognition of the route in the tunnel and causes safety hazards.

Fig 3(c) is the tunnel model at night after the luminescent coating is laid, and the night time is 22:00. The scene is that there is no lighting facility or lighting facility failure in the tunnel at night. At the same time, the inner wall of the tunnel is laid with a luminescent coating, and the road markings are luminescent markings. The brightness is 3.92 cd/m^2^ of No.7 group coating brightness. From Fig 3(c), it can be seen that the visual effect in the tunnel is better after the deployment of luminescent coating, which can guide the line shape and focus the attention of drivers. At the same time, the brightness is within the range of mesopic vision, which is not easy to cause visual fatigue and can be used as emergency lighting in tunnels. When there is no lighting facility, the light source is provided by the lamp, and the luminescent coating absorbs the light source and then releases it in the form of visible light. When the lighting facilities fail, the lights provide a light source, and the light energy absorbed by the luminescent coating before the failure of the lighting facilities is converted and the visible light is released. When lighting is available, it can absorb light from both street lamps and headlights and convert it into visible light to enhance the lighting environment in the tunnel.

### 3.4 Simulation analysis of tunnel light environment based on DIALux

#### 3.4.1 Build a tunnel model.

To study the effect of luminescent coatings applied to the side walls of the tunnel on the illumination level, internal materials with different reflectance ratios were set up and analyzed computationally. At the same time, it is ensured that the other parameters of the tunnel (such as tunnel geometry, lamp type, light distribution curve, luminous flux, lamp arrangement, and reflection coefficients of pavement materials, etc.) are completely fixed. Due to the difficulties in setting up different tests for testing under the existing conditions, DIALux software was used for simulation. Taking the average brightness, average illuminance, uniformity and average brightness improvement rate of the pavement as evaluation indexes, the influence of laying luminescent coating on tunnel lighting effect is simulated and analyzed.

**Fig 4:**
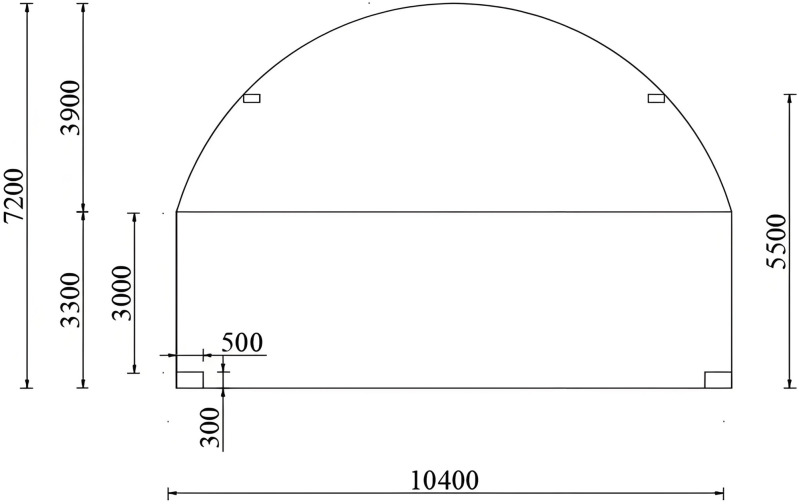
Tunnel cross-section dimension.

**Fig 5:**
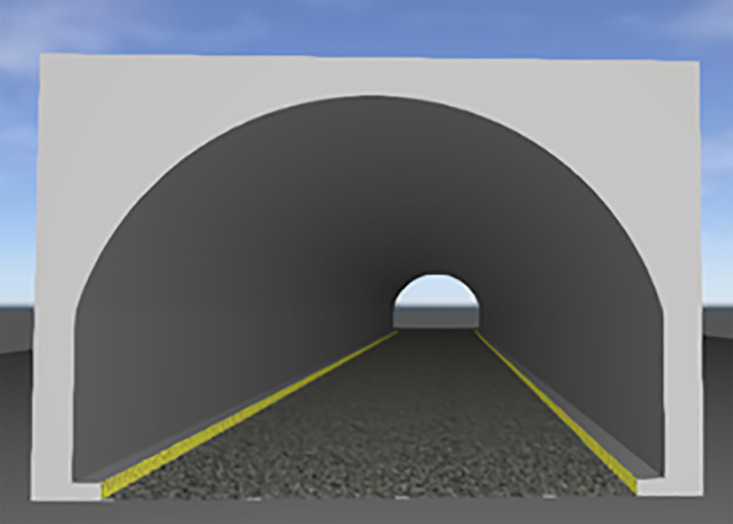
Schematic diagram of tunnel model.

The total length of the tunnel is 1000 m. The design speed is 80 km/h, and the one-way two-lane traffic flow N is 750 veh/(h·ln). The middle section of the tunnel is basic lighting, while not being affected by natural light. Therefore, a length of 100 m in the middle section was intercepted to model the tunnel. The tunnel is 10.4 m wide and 7.2 m high, and the cross-section dimensions are shown in Fig 4, where the data are in mm. Drawing the inner contour of the main tunnel bore in AutoCAD. In DIALux evo, the DWG format tunnel profile file generated by AutoCAD is imported and the 3D model of the tunnel is constructed using the components of the software and Boolean operations. The pavement material is asphalt pavement, and the material information is imported to complete the basic model of the tunnel. The schematic diagram of the tunnel model is shown in Fig 5.

**Fig 6:**
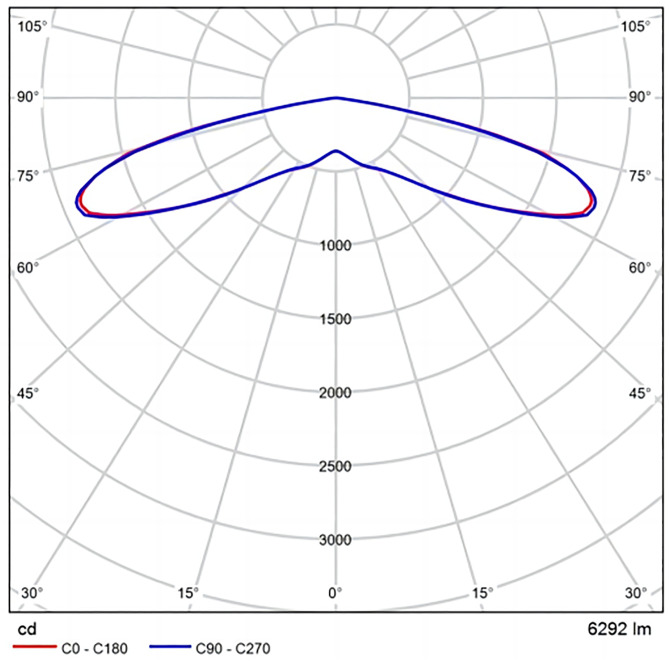
Light distribution curve.

Once the tunnel model has been built, the materials and reflection coefficients need to be set for each part of the tunnel. The purpose of the simulation experiment is to study the improvement effect of the brightness of the road surface after the luminescent coating is arranged in the tunnel. Therefore, except for the need to lay luminescent materials and other materials for the interior decoration of the tunnel side wall, the other parameters are fixed. The pavement material is asphalt pavement, and the reflection ratio is 0.15. The interior wall material of the tunnel is divided into two parts, the roof interior material and the sidewall interior material. Lamps with direct lighting or semi-direct lighting have less luminous flux allocated to the ceiling, and the reflection effect is not obvious. At the same time, the final accumulation of tunnel pollutants is presented in the upper part of the tunnel. Therefore, the layout of high-reflectivity interior materials, after a period of use, the reflectivity will decrease. And the layout of high-reflectivity interior materials will increase the cost. Therefore, dark fire retardant coating is selected to paint on the ceiling, and the reflection ratio is 0.10. To compare the enhancement of pavement brightness after luminescent coating placement, three types of sidewall interior materials were considered: ordinary fire retardant coatings, reflective ceramic tiles, and luminescent coating. Ordinary fire retardant coatings is consistent with the roof material and has a reflectance ratio of 0.10. The reflective ceramic tile is arranged 3 m, and the reflection ratio is 0.58. Luminescent coatings are mainly embodied in the software as a high reflectance ratio, the minimum reflectance ratio of luminescent coatings is 0.85, which is taken in this paper. The lamp adopts the LED lamp of Cooper Lighting manufacturer, with a power of 52.1 w and luminous flux of 6292 lm. The light distribution curve is shown in Fig 6. The layout of the lamp is symmetrically arranged in double rows. The spacing of the lamps is 5 m, the height of the lamps is 5.5 m, and the maintenance coefficient of the lamps is 0.8.

#### 3.4.1 Influence of different materials on the lighting effect of tunnels.

**Fig 7:**
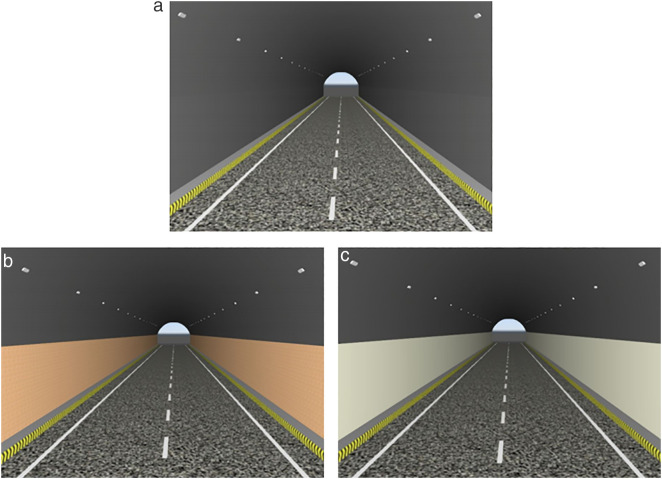
Schematic diagram of the tunnel model with different interior materials. (a) Fire retardant coating (b) Reflective ceramic tiles(c) Luminescent coating.

To simulate the lighting effect of the luminescent coating in the tunnel, three materials of fire retardant coating, reflective ceramic tile, and luminescent coating are laid on the side wall of the tunnel. The tunnel model with lamps arranged symmetrically in two rows, asphalt concrete pavement with markings, and three different interior materials is shown in Fig 7.

Fig 7 shows the schematic diagram of tunnel models with different interior materials. Fig 7(a) shows the fire retardant coating on the side wall of the tunnel, both the side wall and the ceiling are fire retardant coatings, and the reflection ratio is 0.1. Fig 7(b) shows the sidewall arrangement of 3 m reflective ceramic tiles with a reflection ratio of 0.58. Fig 7(c) shows the arrangement of 3 m luminescent coatings. The prepared No.7 group of luminescent coatings was photographed and filled, and the reflection ratio was 0.85. The lights are not shown, only a schematic diagram of the arrangement is given.

In this paper, the evaluation index selects the average brightness, average illumination, uniformity and average brightness improvement rate of the pavement. The average brightness (illuminance) of the pavement is the average value of the brightness (illuminance) of each point measured or calculated on the pre-set point on the pavement, indicating the overall brightness (illuminance) level of the pavement. The tunnel is mainly divided into entrance section, transition section, middle section and exit section. The middle section is the most stable and lowest brightness lighting section in the tunnel. This paper selects the middle section of 100 m for lighting design. The illumination brightness of the middle section will not be affected by the brightness outside the tunnel, only related to the driving speed and traffic flow. The reference value is shown in Table 17.

**Table 17:** Middle section brightness table Lin (cd/m^2^).

Design speed v(km/h)	L_in_(cd/m^2^)		
	One-way traffic N[veh/(h·Ln)]		
	N ≤ 350	350 < N < 1200	N ≥ 1200
	Two-way traffic N[veh/(h·Ln)]		
	N ≤ 180	180 < N < 650	N ≥ 650
120	4.5	6.0	10.0
100	3.0	4.5	6.5
80	1.5	2.5	3.5
60	1.0	1.5	2.0
20-40	1.0	1.0	1.0

The road illumination uniformity index is set to avoid the “zebra effect” inside the tunnel. Road illumination uniformity is a reflection of the uniformity of illumination across the traveling road. The larger the illumination uniformity value, the more uniform the illumination distribution. When the arrangement of lamps is unreasonable, it will lead to uneven distribution of road illumination inside the tunnel. Further, promotes the visual alternating light and dark changes, the emergence of the “zebra effect”. The uniformity of road illumination is calculated according to equation (19). The total uniformity in the middle section of the tunnel is taken according to the traffic volume according to Table 18. When the traffic volume is in the middle value, the value is taken according to the linear internal difference.

**Table 18:** Uniformity of road illumination *U*_*0.*_

design hour volume N[veh/(h·ln)]		*U*_*0*_
One-way traffic	Two-way traffic	
N ≥ 1200	N ≥ 650	0.4
N ≤ 350	N ≤ 180	0.3


$$U_{0}=\frac{E_{\min}}{E_{av}}$$ (19)

Where *U*_*0*_ is the uniformity of road illuminance, *E*_*min*_ is the minimum illuminance of the road, *E*_*av*_ is the average illuminance of the road.

**Fig 8:**
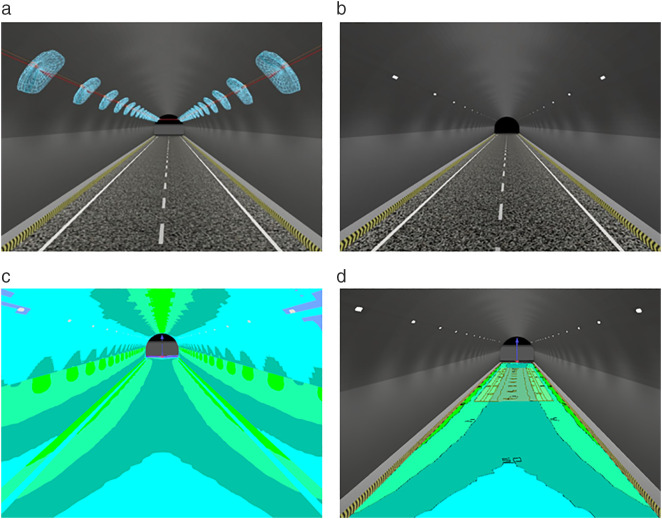
Tunnel-related model when fire retardant coating is laid on the side wall Tunnel. (a) lighting schematic diagram (b)Lighting simulation effect diagram (c) Illumination false color chart (d) Road illumination distribution map.

Fig 8 shows the tunnel-related model of fire retardant coating on the side wall. Table 19 lists the simulation data when the fire retardant coatings were deployed.

**Table 19:** Simulation data for the deployment of fire retardant coating.

Index	Mean value	Minimum value	Maximum value	Uniformity
Illuminance/lx	74.4	34.3	108	0.461
Brightness/cd/m^2^	3.62	1.67	5.27	0.461

**Fig 9:**
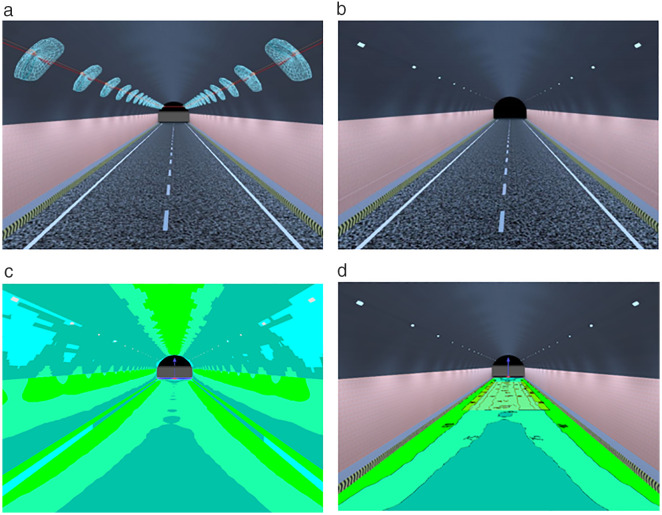
Tunnel-related model when reflective ceramic tiles are laid on the side wall. a)Tunnel lighting schematic diagram (b) Lighting simulation effect diagram (c) Illumination false color chart (d) Road illumination distribution map.

Fig 8 is the tunnel model when the fire retardant coating is laid on the side wall. Fig 8(a) shows the tunnel lighting model, and the light distribution curve of the lamps and lanterns is set up, which can be seen in the position of the lamps and the effect of the lamps and lanterns after the effect diagram. Fig 8(b) shows the overall effect of the tunnel model, showing the illumination effect after the deployment of lights. Fig 8(c) shows an illumination false color chart reflecting the tunnel luminaire deployment and the illuminance inside the tunnel under that luminaire deployment condition. Different colors represent different values, and it can be seen that the illumination distribution is uniform. Fig 8(d) is an illuminance distribution diagram showing the distribution of illuminance in the calculation area for this luminaire deployment. At the same time, combined with Table 19, it can be concluded that the minimum illuminance is 34.3 lx and the maximum illuminance is 108 lx. The uniformity is higher than the standard value, indicating that the uniformity is good. The average brightness of the pavement in the calculation area is 3.62 cd/m^2^, which is in the mesopic visual brightness range and will not cause visual fatigue. Fig 9 gives a model of 3 m reflective ceramic tiles laid on the side walls. It can guide the alignment through the reflective ceramic tiles on the side wall, remind the driver to observe the alignment, and ensure the safety of driving. The simulated data are shown in Table 20.

**Table 20:** Simulated data for the deployment of reflective ceramic tiles.

Index	Mean value	Minimum value	Maximum value	Uniformity
Illuminance/lx	87.5	40	127	0.457
Brightness/cd/m^2^	4.27	1.95	6.22	0.457

From Fig 9 and Table 20, it can be seen that when 3 m reflective ceramic tiles are laid on the side walls, the illuminance as well as the brightness is increased compared to the laying of fire-retardant coating. The mean brightness was 4.27 cd/m^2^ and the mean illuminance was 87.5 lx, with little change in uniformity. It shows that there is an overall improvement in the illumination of the pavement after the deployment of reflective ceramic tiles. The improvement in the overall lighting effect is mainly due to the increased reflection ratio of the sidewall reflective tiles. As the reflectance ratio increases, the brightness and illuminance will increase to some extent. At the same time, the deployment of reflective ceramic tiles in the tunnel has a certain degree of inducement, to provide motorists with information about the direction of the road ahead, the line shape, and so on. It also improves driving safety.

**Fig 10:**
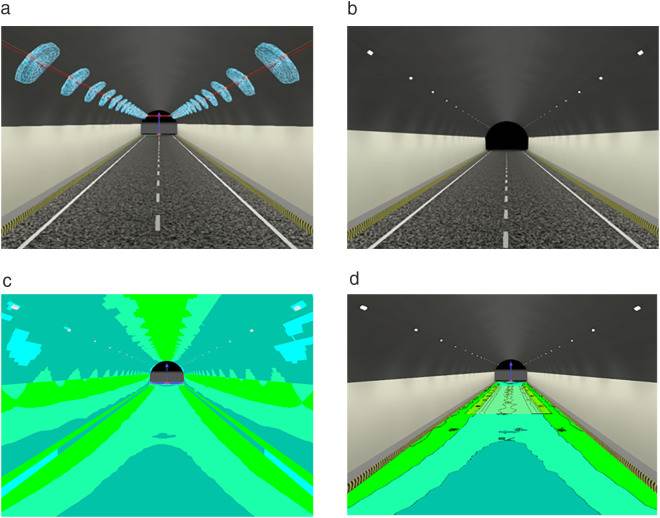
Tunnel-related model when the luminescent coating is laid on the side wall Tunnel. (a) lighting schematic diagram (b) Lighting simulation effect diagram (c) Illumination false color chart (d) Road illumination distribution map.

Fig 10 shows the tunnel related model of luminescent coating on the side wall. The simulated data are shown in Table 21.

**Table 21:** Simulation data for deployment of luminescent coating.

Index	Mean value	Minimum value	Maximum value	Uniformity
Illuminance/lx	91	41.7	132	0.458
Brightness/cd/m^2^	4.43	2.03	6.43	0.458

As can be seen from Fig 10 and Table 21, the illuminance as well as the brightness is increased when 3 m of luminescent coating is laid on the side walls. Since luminescent coatings are high-reflectivity materials, the overall lighting effect inside the tunnel will be better than ordinary materials and low-reflectivity materials. The arrangement of luminescent coating can assist lighting when it is arranged in the middle section of the tunnel. At the same time, drivers are prompted to see the road surface to ensure driving safety. Table 22 shows the simulation data under different conditions.

**Table 22:** Simulation test data under different conditions.

Working condition	Average illumination	Uniformity	Average brightness	Average brightness increase rate
fire retardant coating	74.4lx	0.461	3.62 cd/m^2^	0
reflective ceramic tiles	87.5lx	0.457	4.27 cd/m^2^	18.0%
luminescent coating	91lx	0.458	4.43 cd/m^2^	22.3%

Changing only the sidewall interior material has an effect on the quality of tunnel lighting under the same conditions. The tunnel with luminescent coating has obvious advantages. The average illuminance value is increased from 74.4 lx to 91 lx, and the increase rate is 22.3%. The brightness of the middle section of the tunnel is affected by the driving speed and traffic flow. The design speed is 80 km/ h, and the one-way traffic flow is 750 veh/(h·ln), according to Table 17. The average brightness of the middle section should be greater than 2.5 cd/m^2^, and the three schemes all meet the design requirements of the tunnel. At the same time, the average brightness of the luminescent coating is 4.43 cd/m^2^. That is, the average brightness under the three conditions is in the brightness range of mesopic vision (10^-3^-5 cd/m^2^), and the cone cells and rod cells of the human eye all act. Relevant research shows that when the human eye is in the middle vision, the visual effect is the best. After laying reflective ceramic tiles on the side wall, the average brightness improvement rate is 18%. After the layout of luminescent coatings, the average brightness increase rate is 22.3%, and the effect is more obvious. That is, the arrangement of luminescent coatings in the middle section of the tunnel can assist with lighting, and the lighting effect in the tunnel can be improved as a whole. This ensures that drivers can see the road more clearly in the middle section, where only basic lighting is available, to ensure travel safety. At the same time, the laying of luminescent coating on the side wall can also guide the direction and alignment of the road in front of the road, which has a certain induction effect. The luminescent coating can absorb the light of the lighting facilities in the tunnel and the light source of the headlights. Releases light that is recognizable to the human eye, improving driver recognition of the tunnel environment. In addition, in the event of power outages and other sudden emergencies, the luminescent coating deployed can be used as emergency lighting to guide people to escape.

## 4 Conclusion

In this study, the luminescence performance and colorimetric characteristics of long afterglow luminescent coatings with different ratios were investigated. Comprehensive evaluation of luminescent coatings was carried out by orthogonal test design combined with CW-VIKOR. Analog simulation analysis of the best performing luminescent coatings by Twinmotion and DIALux. Based upon the discussion of the results above, the conclusions are as follows:

(1) Long afterglow luminescent coating luminescence performance is mainly related to the luminescent powder dosage. When the dosage is controlled at 20% −30%, the higher the luminescent powder doping, the better the luminescence performance.
(2) The comprehensive evaluation by CW-VIKOR shows that the performance of No.7 coating is the best. That is, when the dosage of luminescent powder is 30%, the dosage of glass powder is 8%, and the dosage of titanium dioxide is 4%, the luminescent coating has the best luminescence performance and colorimetric characteristics.
(3) Visual simulation was performed using Twinmotion. It is shown that the application of the luminescent coating in tunnels can guide the line shape and ensure the safety of traveling.
(4) The lighting of luminescent coating in the tunnel is quantitatively evaluated by DIALux. When 3 m of luminescent coating was applied to the sidewalls, the average pavement brightness was improved by 22.3% compared to that when fire retardant coating was applied.

## References

[1] Kuang Q, Hou X, Du C, Wang X, Gao D. Recent advances in the anti-counterfeiting applications of long persistent phosphors. Phys Chem Chem Phys. 2023;25(27):17759–68. doi: 10.1039/d3cp01818k 37377090

[2] Thomas NM, Anila EI. Synthesis and Characterisation of SrAl2O4: Eu3+ Orange-Red Emitting Nanoparticles. J Fluoresc. 2024;34(3):1161–9. doi: 10.1007/s10895-023-03351-8 37490253

[3] Zhang F, Xie Y, Zhao X. Aluminate long afterglow luminescent materials in road marking field research progress and development: A review. Buildings. 2024;14(7):2152.

[4] Rojas-Hernandez RE, Rubio-Marcos F, Rodriguez MÁ. Long lasting phosphors: SrAl2O4: Eu, Dy as the most studied material. Renewable and Sustainable Energy Reviews. 2018;81:2759–70.

[5] He R, Liang YP, Xie RS. Research progress in application of strontium aluminate long afterglow luminescent materials in road markings. Journal of Chang’an University (Natural Science Edition). 2022;42(3):1–13.

[6] Nance J, Sparks TD. From streetlights to phosphors: A review on the visibility of roadway markings. Progress in Organic Coatings. 2020;148:105749.

[7] Nance J, Sparks TD. Comparison of coatings for SrAl2O4: Eu2, Dy3 powder in waterborne road striping paint under wet conditions. Progress in Organic Coatings. 2020;144:105637.

[8] Yang X, et al. Mechanical properties, luminescent properties, and durability of solvent-free polyurethane-based phosphorescent road markings on asphalt pavements. Construction and Building Materials. 2024;414:135053.

[9] Xuejuan C, Bailin S, Yongjie D. Preparation and properties of high-brightness long-afterglow luminescent paint. Electroplating & Finishing. 2021;40(8).

[10] Bi Y, Pei J, Chen Z. Preparation and characterization of luminescent road-marking paint. International Journal of Pavement Research and Technology. 2021;14:252–8.

[11] Kim W-J, Lee T-K. Psychophysiological Response According to the Greenness Index of Subway Station Space. Sensors (Basel). 2021;21(13):4360. doi: 10.3390/s21134360 34202277 PMC8272074

[12] Liang B, Cui L, Chen W. S/P value-based optical performance study of light-storing and reflective side wall applied material for auxiliary energy-saving lighting in tunnels. Modern Tunnelling Technology. 2016;53:196–201.

[13] Qiyao G, Jialong D, Yuanyuan Z. Hybrid energy harvesting solar cells―from principles to applications. Progress in Chemistry. 2023;35(2):318–29.

[14] Peng Y, Tao L, Huimei Z. Simulation design of lighting system for Nepal Tanahu HPP. In: 2024 7th International Conference on Electronics Technology (ICET), 2024. 567–71.

[15] Liang B, He S, Liu H. Tunnel lighting calculation model based on bidirectional reflectance distribution function: considering the dynamic changes in light transmittance in road tunnels. Tunnelling and Underground Space Technology. 2023;140:105313.

[16] Shan B, Yang X, Cao X. Preparation of high-luminescent materials and application of luminescent coatings in road engineering. Journal of Materials in Civil Engineering. 2022;34(8):04022159.

[17] Kovbasiuk K, Demčák J, Husár J, et al. A Digital Twin for Remote Learning: A Case Study. Design, Simulation, Manufacturing: The Innovation Exchange. Cham: Springer Nature Switzerland, 2023: 379–89.

[18] Chan HM, Chow CW, Hsu LS. Utilizing lighting design software for simulation and planning of machine learning based angle-of-arrival (AOA) visible light positioning (VLP) systems. IEEE Photonics Journal. 2022;14(6):1–7.

[19] Wang C, Yao X, Ma K. A study of carbon emissions during the operational period of an integrated expressway construction station. Sustainability. 2024;16(17).

[20] Thejo Kalyani N, Jain A, Dhoble SJ. Persistent phosphors for luminous paints: A review. Luminescence. 2022;37(4):524–42. doi: 10.1002/bio.4203 35102701

[21] Chen Z, et al. CdSe/CdS: Eu3 @ SrAl2O4: Eu2 , Dy3 phosphor composite shows red emitting luminescence with high brightness and long lifetime based on efficient PRET and color superposition. Inorganic Chemistry Communications. 2025;173:113882.

[22] Khanh TQ, Bodrogi P, Zandi B, Vinh TQ. Brightness perception under photopic conditions: experiments and modeling with contributions of S-cone and ipRGC. Sci Rep. 2023;13(1):14542. doi: 10.1038/s41598-023-41084-7 37666893 PMC10477289

[23] Casagrande C, Nogueira F, Salmento M. Efficiency in street lighting projects by employing LED luminaires and mesopic photometry. IEEE Latin America Transactions. 2019;17(06):921–9.

[24] Zele AJ, Cao D. Vision under mesopic and scotopic illumination. Front Psychol. 2015;5:1594. doi: 10.3389/fpsyg.2014.01594 25657632 PMC4302711

[25] Xue Z, Wan X, Wang X, Qin X, Song K. Prediction model for laser marking colors based on color mixing. Opt Express. 2024;32(15):26052–68. doi: 10.1364/OE.525740 39538479

[26] Gaur S, Dosapati S, Tawalare A. Stakeholder assessment in construction projects using a CRITIC-TOPSIS approach. Built Environment Project and Asset Management. 2023;13(2):217–37.

[27] Tronnebati I, Jawab F, Frichi Y, et al. Green Supplier Selection Using Fuzzy AHP, Fuzzy TOSIS, and Fuzzy WASPAS: A Case Study of the Moroccan Automotive IndustryJ. Sustainability, 2024, 16(11): 4580.

[28] Mengjie Z, Yuzhong Y. Study on evaluation of coal mine hidden danger investigation and management capability based on combination weight-gray VIKOR. Mining Safety & Environmental Protection. 2023;50(6):141–6.

[29] Shukla AK, Muhuri PK. A novel deep belief network architecture with interval type-2 fuzzy set based uncertain parameters towards enhanced learning. Fuzzy Sets and Systems. 2024;477:108744.

[30] Nawaz M, Adeel A, Akram M. Risk evaluation in failure mode and effect analysis: AHP-VIKOR method with picture fuzzy rough number. Granular Computing. 2024;9(3):69.

